# Resequencing and variation identification of whole genome of the *japonica* rice variety "Longdao24" with high yield

**DOI:** 10.1371/journal.pone.0181037

**Published:** 2017-07-17

**Authors:** Shukun Jiang, Shichen Sun, Liangming Bai, Guohua Ding, Tongtong Wang, Tianshu Xia, Hui Jiang, Xijuan Zhang, Fengming Zhang

**Affiliations:** Cultivation and Farming Research Institute, Heilongjiang Academy of Agricultural Sciences, Harbin, P.R.China; Institute of Genetics and Developmental Biology Chinese Academy of Sciences, CHINA

## Abstract

*Japonica* rice mainly distributes in north of China, which accounts for more than half of the total *japonica* rice cultivated area of China. High yield, good grain quality and early heading date were the main breeding traits and commercial property in this region. We performed re-sequencing and genome wide variation analysis of one typical northern *japonica* rice variety Longdao24 and its parents (Longdao5 and Jigeng83) using the Illumina sequencing technology. 53.17 G clean bases were generated and more than 96.8% of the reads were mapped to the genomic reference sequence. An overall average effective depth of 43.67 × coverage was achieved. We identified 420,475 SNPs, 95,624 InDels, and 14,112 SVs in Longdao24 genome with the genomic sequence of the *japonica* cultivar Nipponbare as reference. We identified 361,117 SNPs and 81,488 InDels between Longdao24 genome and Longdao5 genome. We also detected 428,908 SNPs and 97,209 InDels between Longdao24 genome and Jigeng83 genome. Twenty-two yield related genes, twenty-two grain quality related genes and thirty-nine heading date genes were analyzed in Longdao24. The alleles of *Gn1a*, *EP3*, *SCM2*, *Wx*, *ALK*, *OsLF* and *Hd17* came from the female parent Longdao5. The other alleles of *qGW8*, *SSIVa*, *SBE3*, *SSIIIb*, *SSIIc*, *DTH2*, *Ehd3* and *OsMADS56* came from the male parent Jigeng83. These results will help us to research the genetics basis of yield, grain quality and early heading date in northern rice of China.

## Introduction

Given continuing population growth and increasing competition for arable land between food and energy crops, the next century may witness serious global food shortage problems. Thus, there is a need for an increase in rice yield, because rice is the world’s most important staple food crop. To substantially increase rice yield, Japan initiated a super high-yielding rice breeding program in 1981 that was targeted to raise rice yield 50% within 15 years. However, this target has not yet been realized [1, 2]. Next, the International Rice Research Institute (IRRI) launched a new plant-type breeding program to develop super rice in 1989, with the target of developing a super rice. However, this target was not reached either [1–3]. In order to meet the food demand required by the Chinese people in the 21st century, a program to breed super rice through combining morphological improvement and the utilization of inter-subspecific heterosis was set up by the Ministry of Agriculture and the Ministry of Science and Technology in 1996 and 1997, respectively [1, 2, 4]. Today, nearly 80 super rice varieties have been released and some of them show high grain yields of 12–21 t/hm^2^ in field experiments [4]. The core of super rice breeding is an effective use of germ plasma resources and favorable genes. For example, most of the northern *japonica* super rice varieties have *DEP1* gene, which controlled large erect panicle [5, 6]. Although a large progress about the genetic basis for yield, grain quality and heading date of super rice had achieved [5–7], but the molecular mechanism is still poorly understood.

To meet the global food demands, achievements in molecular genetics of complex traits in rice will be important for increasing yield in the post-genomics era [8]. The draft genomic sequences of two rice subspecies, *japonica* (Nipponbare) and *indica* (93–11), were released [9, 10]; and later, the final genome sequence of Nipponbare was completed by International Rice Genome Sequencing Project [11]. These achievements provided a high quality reference genome for re-sequencing rice genomes using high-throughput sequencing technologies [12]. The widely used high-throughput sequencing technologies are also known as next-generation sequencing (NGS) technology. The NGS technology has been widely used in rice genomics and molecular breeding studies [13]. These characteristics have enabled researchers to perform accurate genetic polymorphism analysis and discovered important cloned genes of rice variety [14]. Several super rice or its parents including Liangyoupei 9 [15], Shanyou 63 [16] and Shennong 265 [17] has been re-sequenced. However the Heilongjiang super rice varieties such as Longdao 5, Songgeng 9, Longgeng 31 and so on still not be researched. In this study, we re-sequenced a new bred elite *japonica* rice variety, Longdao24, with high quality, super high yield and early heading date in Heilongjiang province. Our results provided several candidates account for the super high yield, good quality and early heading date of rice in Heilongjaing province. The results therefore lay the groundwork for long-term efforts to uncover important genes and for future variety improvement.

## Materials and methods

### Plant materials

**Fig 1:**
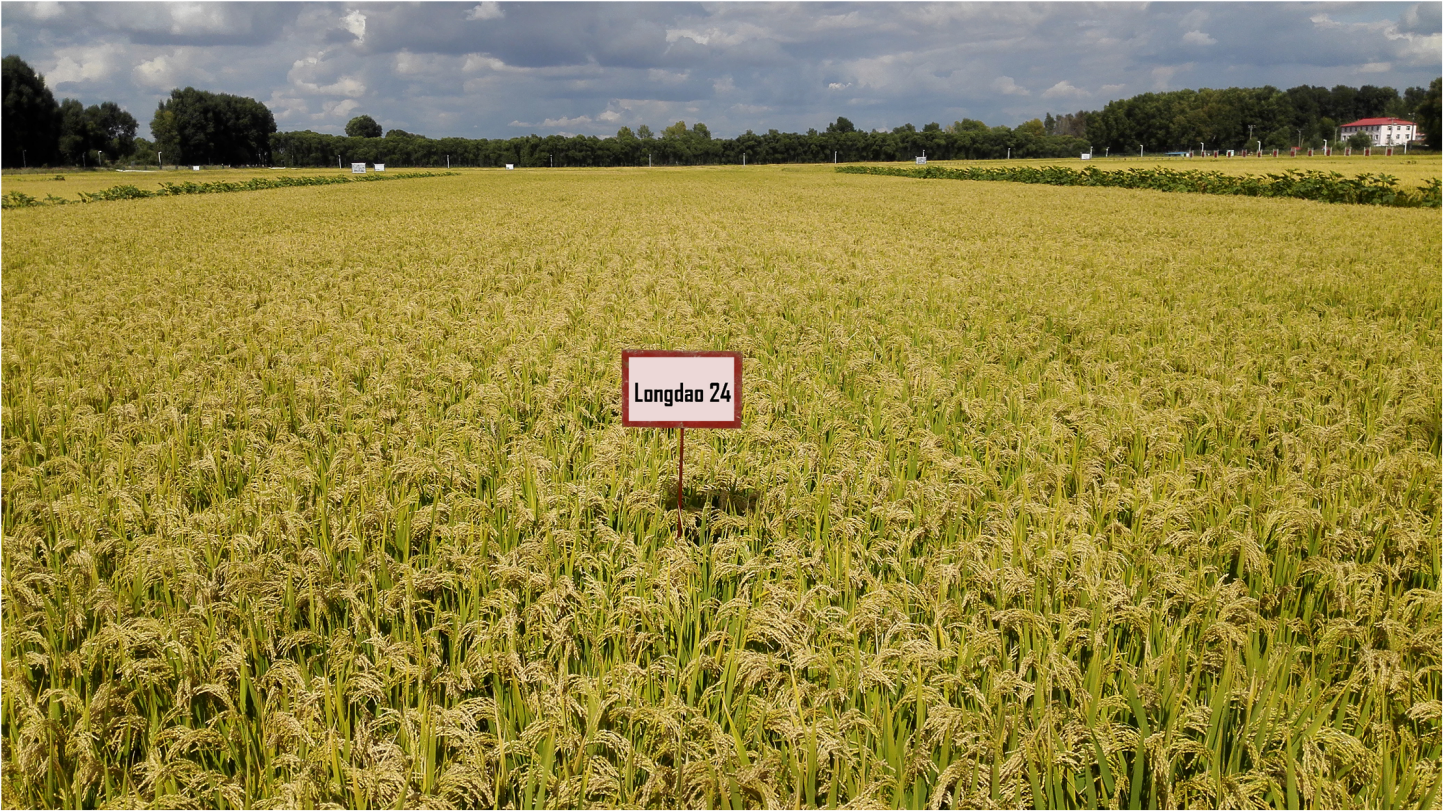
The field performances of Longdao24.

Longdao24 (Fig 1) was selected from the cross between two super *japonica* rice Longdao5 and Jigeng83. Longdao5 is a leading cultivar widely cultivated in the south and east part of Heilongjiang province and has high resistance to disease and super high yield. Jigeng83 is a leading cultivar widely cultivated in the middle part of Jilin province and has super high yield.

### DNA isolation and genome sequencing

Genomic DNA was prepared from bulk harvested leaves of a single young plant of Longdao24, Longdao5 and Jigeng83 using a modified CTAB method [18]. Whole genome re-sequencing of the grape lines was performed on an Illumina HiSeq4000™ by Biomarker Technologies (Beijing, China). The procedure was performed as the standard Illumina protocol, including sample preparation and sequencing as follows: quantified DNA was extracted and DNA fragments were obtained using the ultrasonic wave technique, which was purified using the QIA quick PCR kit. End repair was performed with poly-A on the 3´ ends, then the adaptors were ligated, clusters were generated, agarose gel electrophoresis was used to select fragments, and PCR amplification was performed. Sequencing was performed by establishing a library with Illumina HiSeq4000™. The short reads were aligned using the Burrows–Wheeler transformation [19]. To ensure high quality, Low-quality reads (<20), reads with adaptor sequence and duplicated reads were filtered, and the remaining high-quality data were used in the mapping.

### Mapping of reads to the reference

The clean sequencing reads were aligned to the temperate *japonica* Nipponbare reference genome—the unified-build release Os-Nipponbare-Reference-IRGSP-1.0 [20], using the BWA software under the default parameters with a small modification. The alignment results were then merged and indexed as BAM files [21]. Average sequencing depth and coverage were calculated using the alignment results. The mapped reads were then used to detect SNPs, InDels and SVs polymorphisms.

### Detection of SNPs, InDels and SVs polymorphisms

Firstly, we used GATK tools software [22] to detect SNPs and InDels. Secondly, SVs were detected using BreakdancerMax.pl software [23] with its default parameters. In our result, the types of SVs include insertion (INS), deletion (DEL), inter chromosomal translocation (CTX), deletion including insertion (IDE), intra chromosomal translocation (ITX) and inversion (INV). And in our analysis, InDels were defined as the insertion or deletion the length of which was from 1 to 10 bp.

### Annotation of SNPs, InDels and SVs

The annotation of SNPs and Indels were performed by SnpEff software [24]. The localization of SNPs, InDels and SVs were based on the annotation of reference genome databases. The polymorphisms in the gene region and other genome regions were annotated as genic and intergenic. The SNPs, InDels and SVs were classified according to their localization. SNPs in the CDS were further separated into synonymous and non-synonymous. The functional annotation data were achieved by blast each gene including non-synonymous SNPs or SVs to NR [25], SwissProt [25], GO [26], COG [27] and KEGG [28] database.

## Results

### Genome sequencing of Longdao24 and its parents

Genome sequencing of Longdao24, Longdao5 and Jigeng83 yielded 64.19, 53.93 and 70.60 million reads, most of them being 300 bp paired end reads. After cleaning, 19.17 G (Longdao24), 16.07 G (Longdao5) and 21.11 G (Jigeng83) clean bases were generated and 96.80%, 99.27% and 98.99% of the reads were mapped to the genomic reference sequence. The overall effective depth coverage of Longdao24, Longdao5 and Jigeng83 was 47 ×, 32 × and 52 ×, respectively. The 1 ×, 5 × and 10 × coverage ratio of Longdao24 to the reference genome was 97.87%, 96.11% and 95.08%, respectively. The 1 ×, 5 × and 10 × coverage ratio of Longdao5 to the reference genome was 97.21%, 95.44% and 93.19%, respectively. The 1 ×, 5 × and 10 × coverage ratio of Jigeng83 to the reference genome was 98.40%, 97.32% and 96.63%, respectively. The state information of Longdao24 and its parent were showed in Table 1.

**Table 1:** The re-sequencing state information of Longdao24 and its parent.

	Longdao24	Longdao5	Jigen83
Raw_Reads	64192132	53928143	70604131
Clean_Reads	63958731	53582058	70340507
Clean_Base	19178366460	16070000000	21108274430
Mapped(%)	96.80	99.27	98.99
Q30(%)	94.86	90.82	94.97
Ave_depth	47	32	52
Cov_ratio_1X(%)	97.87	97.21	98.4
Cov_ratio_5X(%)	96.11	95.44	97.32
Cov_ratio_10X(%)	95.08	93.19	96.63

### Frequency distributions of different variation

**Fig 2:**
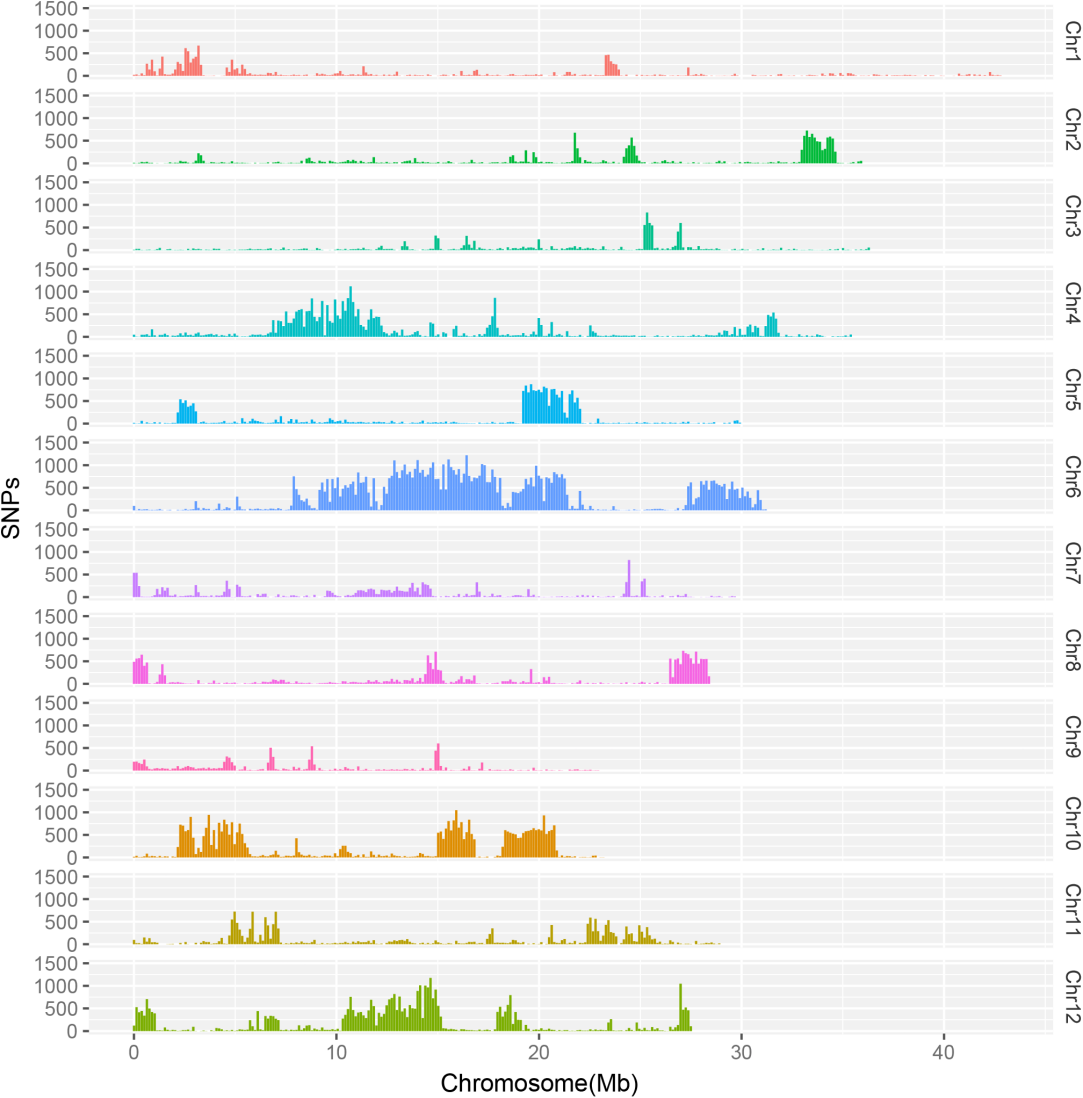
The frequencies of SNPs for Longdao24 at a 100 kb sliding window along each chromosome.

**Fig 3:**
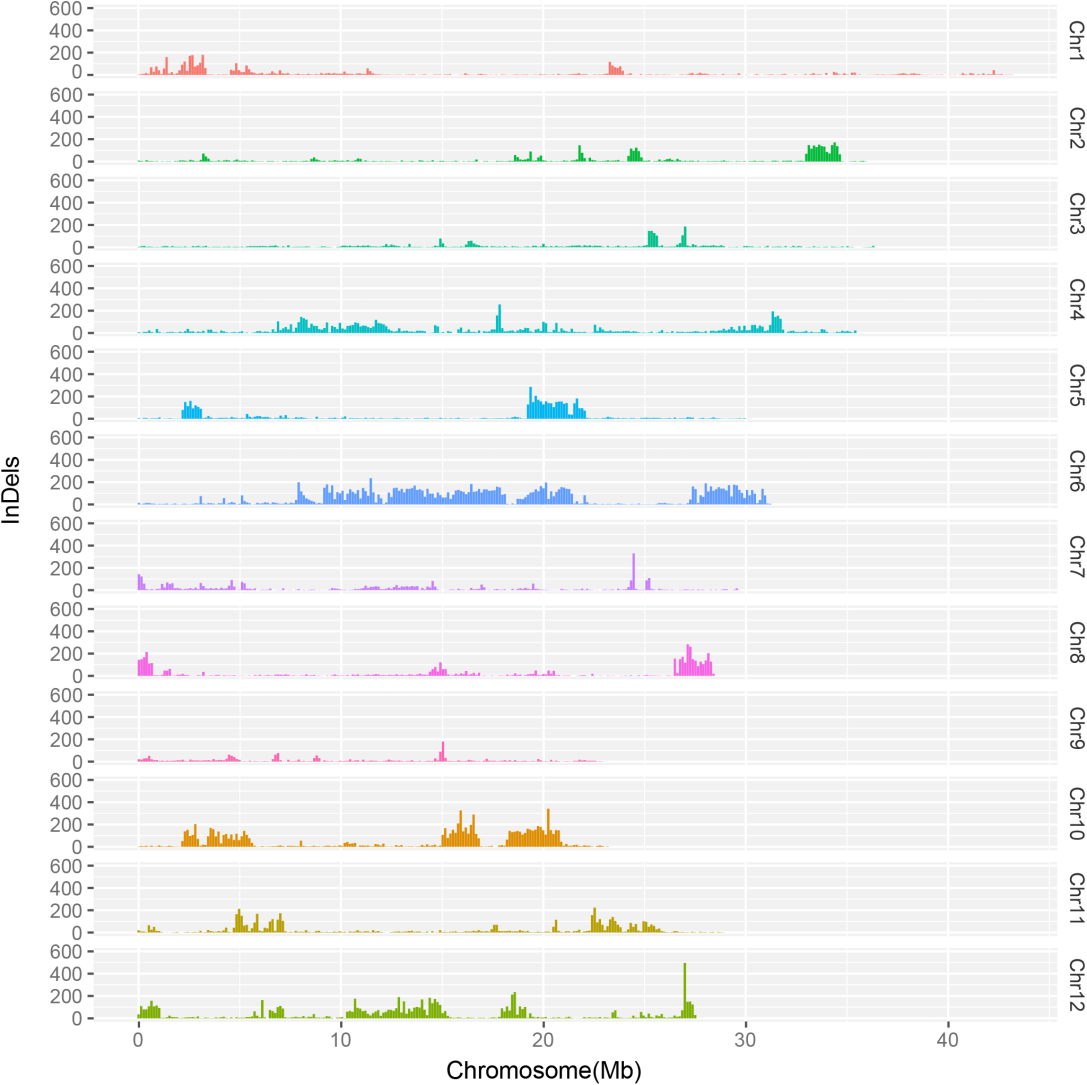
The frequencies of Indels for Longdao24 at a 100 kb sliding window along each chromosome.

**Fig 4:**
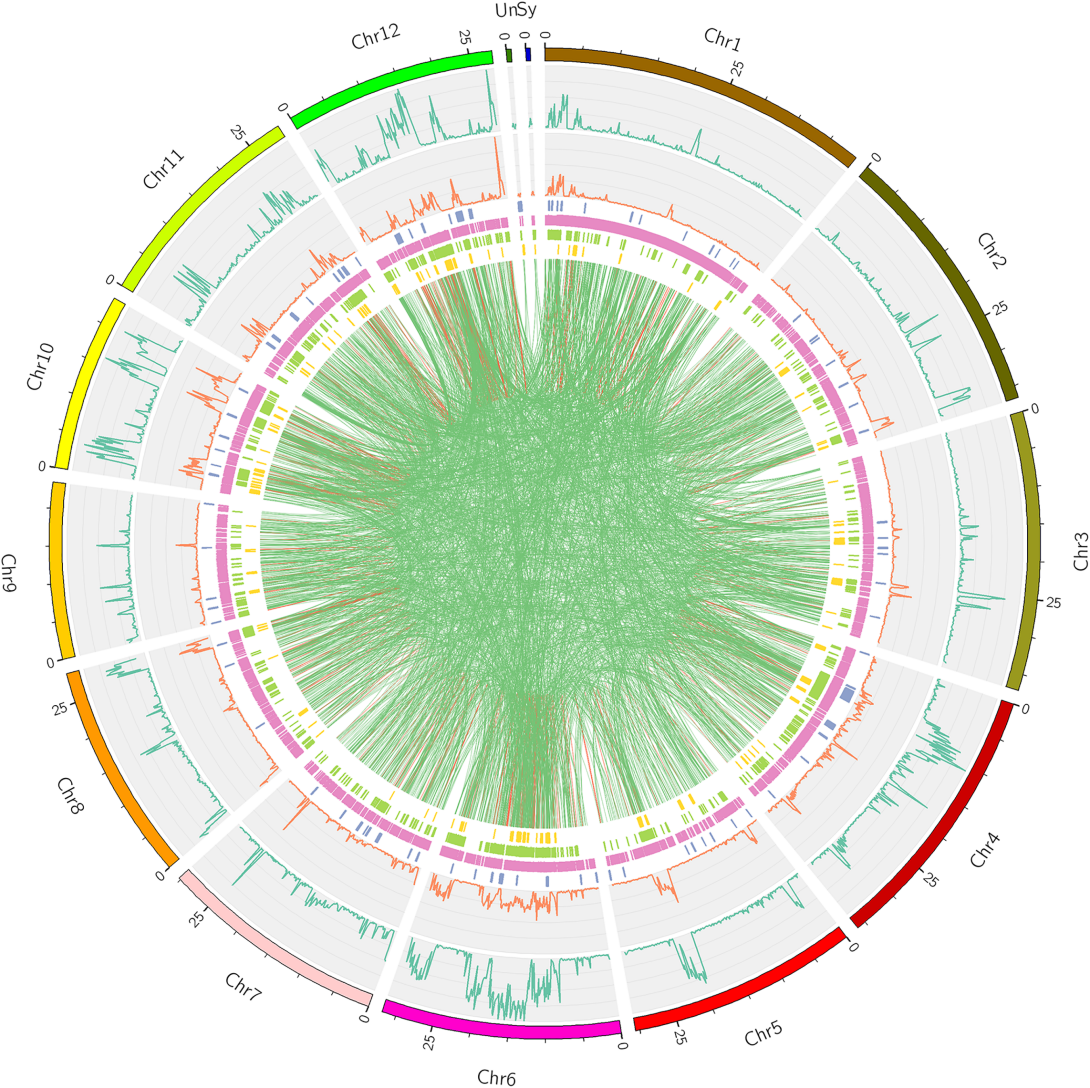
Summary of different variation between Longdao24 and reference variety Nipponbare. From the outside to the inside in turn is chromosome location, distribution of SNP density, distribution of InDel density, Copy number variation (CNV), Structure Variation (Insertion (INS), Deletion (DEL), Inversion (INV), Intra-chromosomal Translocation (ITX, red line) and Inter-chromosomal Translocation (CTX, green line)).

We then examined genome-wide variations. 420,475 SNPs, 95,624 InDels, and 14,112 SVs were yielded from the Longdao24 genome. The frequency distributions of SNPs and InDels on Longdao24 genome were showed in Fig 2 and Fig 3. We detected ten abundant SNPs high region on chr 2(33–35 Mb), chr 4(6.5–12 Mb), chr 5(19-22Mb), chr 6(10-22Mb, 27-31Mb), chr 8(21.5–23 Mb), chr 10(2–6 Mb, 15-16Mb, 17–21 Mb) and chr12 (10–15 Mb). Moreover, the most abundant InDels region were identified on chr 2(33–35 Mb), chr 5(19-22Mb), chr 6(10-22Mb, 27-31Mb), chr 8(21.5–23 Mb) and chr 10(2–6 Mb, 15-16Mb, 17–21 Mb). Several chromosomal loci in the SNP high region were also found covered by the InDel high regions (Figs 2 and 3), which might suggest that those regions were the most polymorphic. 361,117 SNPs and 81,488 InDels were detected between the Longdao24 genome and Longdao5 genome. 428,908 SNPs and 97,209 InDels were identified between the Longdao24 genome and Jigeng83 genome. Frequency distributions of all different variation of Longdao24 genome was showed on Fig 4 via circos software.

### Characteristics of SNPs, InDels and SVs

**Fig 5:**
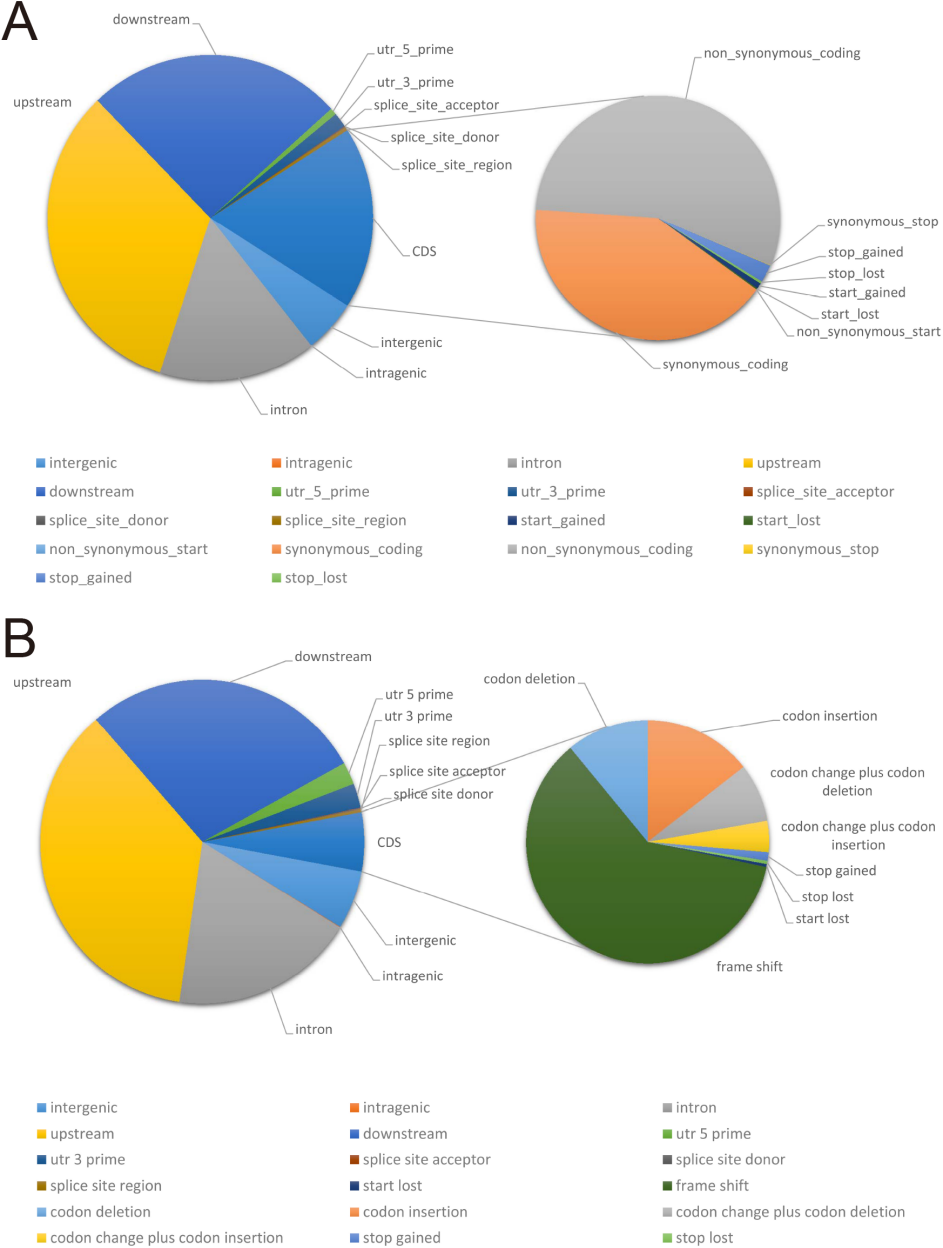
Summary of SNPs and Small Indels detected in Longdao24.

22,250, 65,858, 137,848 and 107,288 SNPs were found in intergenic region, intron region, upstream region and downstream region respectively. 9,054 SNPs were located in the 5’UTR and 3’UTR region. 1,958 SNPs were detected near the splice site. A total of 76,214 SNPs were located in the CDS regions, among which 31,308 were synonymous coding sequences (Syn CDS) and 42,115 were non-synonymous coding sequences (Nonsyn CDS) (Fig 5A).

5,623, 17,609, 34,717 and 27,132 InDels were found in intergenic region, intron region, upstream region and downstream region respectively. 4,465 InDels were located in the 5’UTR and 3’UTR region. 426 InDels were detected near the splice site. A total of 5,602 InDels were located in the CDS regions, among which 3,401 caused frame shift and 2,092 caused codon change (Fig 5B).

A total of 14,112 Structure Variation (SVs) were detected in Longdao24 including 7,795 Insertion (INS), 3,273 Deletion (DEL), 217 Inversion (INV), 317 Intra-chromosomal Translocation (ITX) and 2396 Inter-chromosomal Translocation (CTX). A total of 11,285 SVs were located in the CDS regions, among which 2,969 were detected in exon, 1,697 were located in intron and 6,619 were located in intergenic region. And 2,677 Copy number variation (CNVs) were also detected in Longdao24.

### Variation annotation and gene categories

**Fig 6:**
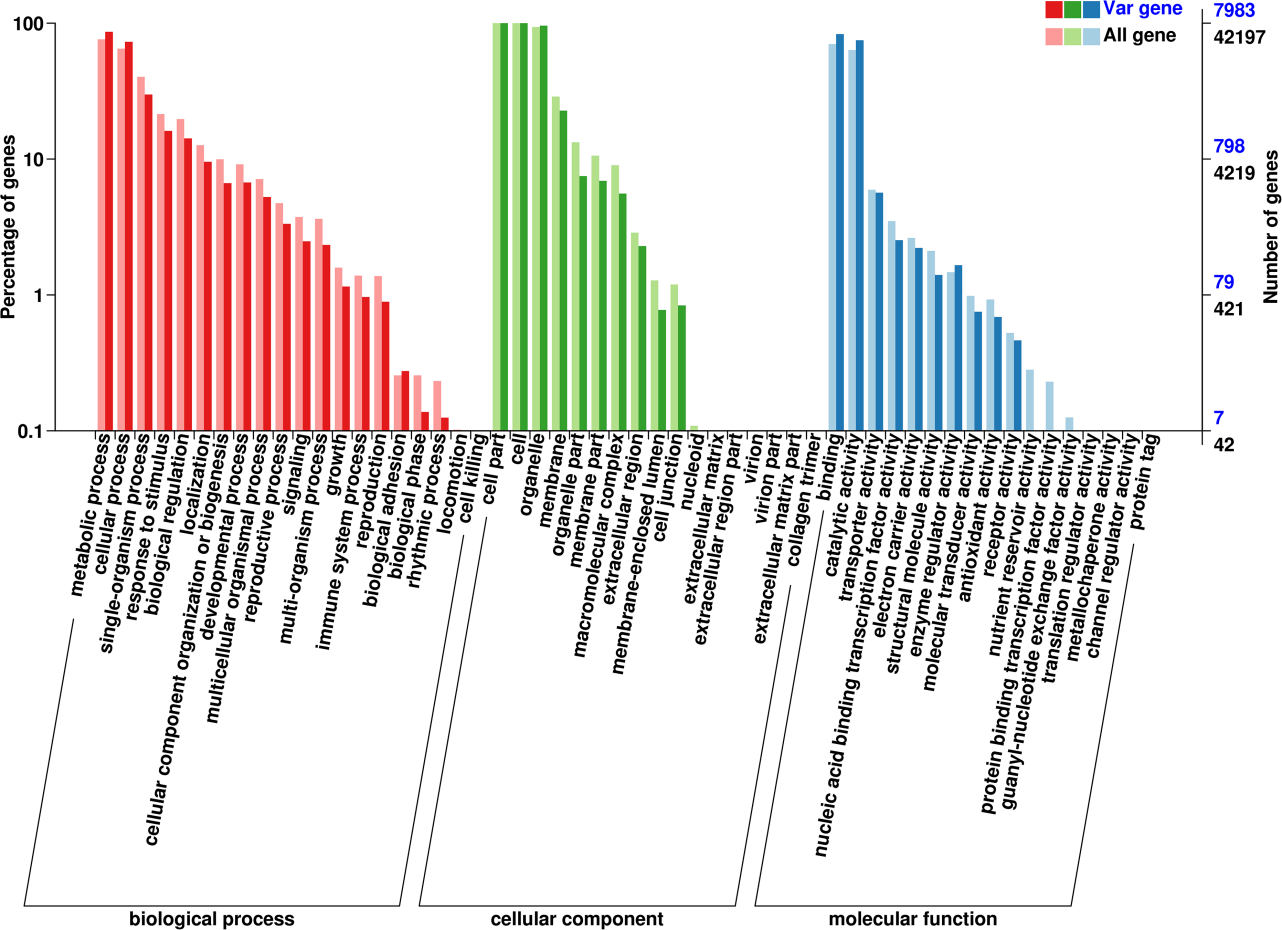
GO classify for differential UniGenes in Longdao24.

16,831 variations including 10,615 non-synonymous SNP mutations, 3,727 frame shifts and 2,489 SVs caused by indels, transversions, and transitions in 12,772 genes might influence the expression of the relevant protein. All tested and functional annotated genes were classified into gene ontology (GO) categories (Fig 6), which mainly divided the genes into three categories: cellular components (20 groups), molecular function (17 groups), and biological process (17 groups). Classification of gene variations compared with COG database by blast showed that most variations (1,935) were identified in replication, recombination and repair progress. Compared with the reference genome, most variations was screened in genetic information processing and metabolism.

### Variation analysis on high yield genes

We investigated 22 cloned yield related genes [7] which might explain the high yield of Longdao24. A large number of SNPs and Indels (Table 2) were only detected in the CDS sequence of 4 genes related to high yield among Longdao24, Longdao5 and Jigeng83, such as *Gn1a* (related to spikelet number), *EP3* (related to erect panicle and spikelet number), *SCM2* (related to lodging resistance and spikelet number) and *qGW8* (related to grain size and grain quality). Four non-synonymous SNPs and a codon insertion InDel were identified in *Gn1a* locus. Two non-synonymous SNPs and a codon change + deletion InDel were identified in *SCM2* locus. Three non-synonymous SNPs and a codon deletion InDel were identified in *qGW8* locus. One frame shift InDel were identified in *EP3* locus. The allele of *Gn1a*, *DEP3* and *SCM2* were come from the female parent Longdao5. The allele of *qGW8* were come from the male parent Jigeng83.

**Table 2:** Variations of 22 high yield related genes in Longdao 24.

Genes	RAP NO.	MSU NO.	Variation type	Variation region	chromosome	location	Nipponbare	Longdao24	Longdao5	Jigeng83
*Gn1a*	Os01g0197700	LOC_Os01g10110	SNP: Non Synonymous	Exon	Chr1	5270928	A	C	C	A
			SNP: Non Synonymous	Exon	Chr1	5275176	T	C	C	T
			SNP: Non Synonymous	Exon	Chr1	5275210	C	A	A	C
			INDEL: Codon Insertion	Exon	Chr1	5275281	A	ACGGCGG	ACGGCGG	A
			SNP: Non Synonymous	Exon	Chr1	5275362	C	G	G	C
*Sd1*	Os01g0883800	LOC_Os01g66100	-	-	-	-	-	-	-	-
*qGY2-1*	Os02g0154200	LOC_Os02g05980	-	-	-	-	-	-	-	-
*GW2*	Os02g0244100	LOC_Os02g14720	-	-	-	-	-	-	-	-
*EP3/LP*	Os02g0260200	LOC_Os02g15950	INDEL: Frame Shift	Exon	Chr2	9042161	CCTCTCTCT	CCTCTCT	CCTCTCT	CCTCTCTCT
*OsGRF4/GS2/GL2*	Os02g0701300	LOC_Os02g47280	-	-	-	-	-	-	-	-
*GL3*.*1/qGL3-1/ qGL3/OsPPKL1*	Os03g0646900	LOC_Os03g44500	-	-	-	-	-	-	-	-
*SCM3/OsTB1/FC1*	Os03g0706500	LOC_Os03g49880	-	-	-	-	-	-	-	-
*GIF1/OsCIN2*	Os04g0413500	LOC_Os04g33740	-	-	-	-	-	-	-	-
*SPIKE/LSCHL4/NAL1/GPS*	Os04g0615000	LOC_Os04g52479	-	-	-	-	-	-	-	-
*GS5*	Os05g0158500	LOC_Os05g06660	-	-	-	-	-	-	-	-
*qSW5*	Os05g0187500	LOC_Os05g09520	-	-	-	-	-	-	-	-
*HGW*	Os06g0160400	LOC_Os06g06530	-	-	-	-	-	-	-	-
*TGW6*	Os06g0623700	LOC_Os06g41850	-	-	-	-	-	-	-	-
*GW6a/OsglHAT1*	Os06g0650300	LOC_Os06g44100	-	-	-	-	-	-	-	-
*SCM2/APO1*	Os06g0665400	LOC_Os06g45460	INDEL: Codon change + Codon deletion	Exon	Chr6	27480424	ACCGCCGCCG	A	A	ACCGCCGCCG
			SNP: Non Synonymous	Exon	Chr6	27480778	G	C	C	G
			SNP: Non Synonymous	Exon	Chr6	27481339	T	C	C	T
*GL7/GW7*	Os07g0603300	LOC_Os07g41200	-	-	-	-	-	-	-	-
*DEP2/EP2/SRS1*	Os07g0616000	LOC_Os07g42410	-	-	-	-	-	-	-	-
*OsSPL14/IPA1/WFP*	Os08g0509600	LOC_Os08g39890	-	-	-	-	-	-	-	-
*qGW8/OsSPL16*	Os08g0531600	LOC_Os08g41940	INDEL:critical polymorphism	5'UTR	Chr8	26501191	A	AAGCTGAGCTG	A	AAGCTGAGCTG
			SNP: Non Synonymous	Exon	Chr8	26501458	C	T	C	T
			INDEL: Codon deletion	Exon	Chr8	26501540	TGCG	T	TGCG	T
			SNP: Non Synonymous	Exon	Chr8	26505387	C	A	C	A
			SNP: Non Synonymous	Exon	Chr8	26505658	G	T	G	T
*DEP1/EP1/DN1/qPE9-1/qNGR9*	Os09g0441900	LOC_Os09g26999	-		-	-	-	-	-	-
*TAWAWA1*	Os10g0478000	LOC_Os10g33780	-		-	-	-	-	-	-

### Variation analysis on starch quality genes

We investigated 22 cloned grain quality related genes [29] in Longdao24 (Table 3). A total of fourteen SNPs and one Indels were detected in the CDS sequence of seven grain quality genes among Longdao24, Longdao5 and Jigeng83. Eleven SNPs and one Indels were detected in *GBSSII* (granule-bound starch synthase II), *SSIIIb* (Soluble starch synthase IIIb) and *SSIIc* (Soluble starch synthase IIc) locus between Longdao24 and Nipponbare. The *GBSSII* in Longdao24 had only one SNP. The *SSIIIb* and *SSIIc* of Longdao24 had six SNPs plus one Indel and four SNPs, respectively. The other three SNPs were detected in *SSIVa* and *SBE3* locus among Longdao24, Longdao5 and Jigeng83. The allele of *GBSSII* locus was same among Longdao24, Longdao5 and Jigeng83, but different from Nipponbare. The other four allele of *SSIVa*, *SBE3*, *SSIIIb* and *SSIIc* were come from the male parent Jigeng83.

**Table 3:** Variations of 22 grain quality related genes in Longdao 24.

Genes	RAP NO.	MSU NO.	Variation type	Variation region	Variation chromosome	Variation location	Nipponbare	Longdao24	Longdao5	Jigeng83
*AGPL2*	Os01g0633100	LOC_Os01g44220	-	-	-	-	-	-	-	-
*SSIVa*	Os01g0720600	LOC_Os01g52250	SNP: Non Synonymous	Exon	Chr1	30038156	A	A	G	A
*SBE3*	Os02g0528200	LOC_Os02g32660	SNP: Non Synonymous	Exon	Chr2	19360942	G	G	A	G
			SNP: Non Synonymous	Exon	Chr2	19361347	T	T	C	T
*SSIIb*	Os02g0744700	LOC_Os02g51070	-	-	-	-	-	-	-	-
*AGPL1*	Os03g0735000	LOC_Os03g52460	-	-	-	-	-	-	-	-
*SBE4*	Os04g0409200	LOC_Os04g33460	-	-	-	-	-	-	-	-
*SSIIIb*	Os04g0624600	LOC_Os04g53310	SNP: Non Synonymous	Exon	Chr4	31752654	G	C	G	C
			SNP: Non Synonymous	Exon	Chr4	31755480	C	T	C	T
			SNP: Non Synonymous	Exon	Chr4	31755497	C	A	C	A
			SNP: Non Synonymous	Exon	Chr4	31757436	C	T	C	T
			SNP: Non Synonymous	Exon	Chr4	31757746	G	A	G	A
			SNP: Non Synonymous	Exon	Chr4	31758185	T	G	T	G
			INDEL: Splice Site	Splice Site	Chr4	31758814	G	GA	G	GA
*ISA2*	Os05g0393700	LOC_Os05g32710	-	-	-	-	-	-	-	-
*SSIVb*	Os05g0533600	LOC_Os05g45720	-	-	-	-	-	-	-	-
*AGPL3*	Os05g0580000	LOC_Os05g50380	-	-	-	-	-	-	-	-
*GBSSI/Wx*	Os06g0133000	LOC_Os06g04200	-	-	-	-	-	-	-	-
*SSI*	Os06g0160700	LOC_Os06g06560	-	-	-	-	-	-	-	-
*SSIIa/ALK*	Os06g0229800	LOC_Os06g12450	-	-	-	-	-	-	-	-
*SBE1*	Os06g0726400	LOC_Os06g51084	-	-	-	-	-	-	-	-
*AGPL4*	Os07g0243200	LOC_Os07g13980	-	-	-	-	-	-	-	-
*GBSSII*	Os07g0412100	LOC_Os07g22930	SNP: Non Synonymous	Exon	Chr7	12917914	A	G	G	G
*SSIIIa*	Os08g0191433	LOC_Os08g09230	-	-	-	-	-	-	-	-
*AGPS2*	Os08g0345800	LOC_Os08g25734	-	-	-	-	-	-	-	-
*ISA1*	Os08g0520900	LOC_Os08g40930	-	-	-	-	-	-	-	-
*AGPS1*	Os09g0298200	LOC_Os09g12660	-	-	-	-	-	-	-	-
*ISA3*	Os09g0469400	LOC_Os09g29404	-	-	-	-	-	-	-	-
*SSIIc*	Os10g0437600	LOC_Os10g30156	SNP: Non Synonymous	Exon	Chr10	15673341	C	T	C	T
			SNP: Non Synonymous	Exon	Chr10	15675357	G	A	G	A
			SNP: Non Synonymous	Exon	Chr10	15675473	G	T	G	T
			SNP: Non Synonymous	Exon	Chr10	15680316	C	A	C	A

### Variation analysis on heading date genes

Eight (*DTH2*, *OsLF*, *Hd17*, *Hd3a*, *Hd1*, *Hd2*, *Ehd3* and *OsMADS56*) from 39 heading date genes had 24 variations (19 SNPs and 5 Indels) among Longdao24, Longdao5 and Jigeng83 (Table 4). The only one non-synonymous SNP in *DTH2* allele from Longdao24 was same as Nipponbare and Jigeng83, but different from Longdao5. *Hd1* allele in Longdao24 had 6 non-synonymous SNPs, one frame shift Indel and three long base pare insertion (36 bp, 69 bp and 51 bp). *Hd2*, *Ehd3* and *OsMADS56* in Longdao24 all be identified with 3 non-synonymous SNPs. *OsLF* in Longdao24 all had one non-synonymous SNP and one Indel. Only one non-synonymous SNP was detected in *Hd3a* and *Hd17*. The SNPs and Indels in *Hd3a*, *Hd2* and *Hd1* locus were detected between Nipponbare with the other three varieties. The allele of *OsLF* were come from the female parent Longdao5. The other fourth allele of *DTH2*, *Hd17*, *Ehd3* and *OsMADS56* were come from the male parent Jigeng83.

**Table 4:** Variations of 39 heading date related genes in Longdao 24.

Genes	RAP NO.	MSU NO.	Variation type	Variation region	Variation chromosome	Variation location	Nipponbare	Longdao24	Longdao5	Jigeng83
*OsGI*	Os01g0182600	LOC_Os01g08700	-	-	-	-	-	-	-	-
*OsEF3*	Os01g0566100	LOC_Os01g38530	-	-	-	-	-	-	-	-
*OsLFL1*	Os01g0713600	LOC_Os01g51610	-	-	-	-	-	-	-	-
*OsCCT01*	Os01g0835700	LOC_Os01g61900	-	-	-	-	-	-	-	-
*OsMADS65*	Os01g0922800	LOC_Os01g69850	-	-	-	-	-	-	-	-
*Se13*	Os01g0949400	LOC_Os01g72090	-	-	-	-	-	-	-	-
*OsCOL4*	Os02g0610500	LOC_Os02g39710	-	-	-	-	-	-	-	-
*DTH2*	Os02g0724000	LOC_Os02g49230	SNP: Non Synonymous	Exon	Chr2	30098026	T	T	G	T
*PPS*	Os02g0771100	LOC_Os02g53140	-	-	-	-	-	-	-	-
*rhd1*	Os02g0776900	LOC_Os02g53690	-	-	-	-	-	-	-	-
*HDR1*	Os02g0793900	LOC_Os02g55080	-	-	-	-	-	-	-	-
*Ehd4*	Os03g0112700	LOC_Os03g02160	-	-	-	-	-	-	-	-
*DTH3*	Os03g0122600	LOC_Os03g03070	-	-	-	-	-	-	-	-
*Se14*	Os03g0151300	LOC_Os03g05680	-	-	-	-	-	-	-	-
*OsHAP5C*	Os03g0251350	LOC_Os03g14669	-	-	-	-	-	-	-	-
*OsPRR73*	Os03g0284100	LOC_Os03g17570	-	-	-	-	-	-	-	-
*Hd6*	Os03g0762000	LOC_Os03g55389	-	-	-	-	-	-	-	-
*Hd16*	Os03g0793500	LOC_Os03g57940	-	-	-	-	-	-	-	-
*SPIN1*	Os03g0815700	LOC_Os03g60110	-	-	-	-	-	-	-	-
*OsRR1*	Os04g0442300	LOC_Os04g36070	-	-	-	-	-	-	-	-
*HAF1*	Os04g0648800	LOC_Os04g55510	-	-	-	-	-	-	-	-
*OsLF*	Os05g0541400	LOC_Os05g46370	SNP: Non Synonymous	Exon	Chr5	26880725	C	G	G	C
			INDEL: Frame Shift	Exon	Chr5	26880755	C	CGG	CGG	C
*Hd17*	Os06g0142600	LOC_Os06g05060	SNP: Non Synonymous	Exon	Chr6	2235191	A	G	A	G
*RFT1*	Os06g0157500	LOC_Os06g06300	-	-	-	-	-	-	-	-
*Hd3a*	Os06g0157700	LOC_Os06g06320	SNP: Non Synonymous	Exon	Chr6	2942192	C	G	G	G
*Hd1*	Os06g0275000	LOC_Os06g16370	INDEL: Codon Insertion	Exon	Chr6	9336861	C	37 bp	37 bp	37 bp
			SNP: Non Synonymous	Exon	Chr6	9336938	A	G	G	G
			SNP: Non Synonymous	Exon	Chr6	9336985	G	A	A	A
			INDEL: Codon change + Codon Insertion	Exon	Chr6	9337012	A	70 bp	70 bp	70 bp
			SNP: Non Synonymous	Exon	Chr6	9337021	G	A	A	A
			INDEL: Codon Insertion	Exon	Chr6	9337032	C	52 bp	52 bp	52 bp
			SNP: Non Synonymous	Exon	Chr6	9337148	G	A	A	A
			INDEL: Frame Shift	Exon	Chr6	9338004	CTT	C	C	C
			SNP: Non Synonymous	Exon	Chr6	9338068	A	C	C	C
			SNP: Non Synonymous	Exon	Chr6	9338330	G	A	A	A
*Se5*	Os06g0603000	LOC_Os06g40080	-	-	-	-	-	-	-	-
*Ghd7*	Os07g0261200	LOC_Os07g15770	-	-	-	-	-	-	-	-
*Hd2/Ghd7*.*1*	Os07g0695100	LOC_Os07g49460	SNP: Non Synonymous	Exon	Chr7	29617569	G	C	C	C
			SNP: Non Synonymous	Exon	Chr7	29623803	G	A	A	A
			SNP: Non Synonymous	Exon	Chr7	29628500	T	C	C	C
*Ehd3*	Os08g0105000	LOC_Os08g01420	SNP: Non Synonymous	Exon	Chr8	274460	G	C	G	C
			SNP: Non Synonymous	Exon	Chr8	276145	G	A	G	A
			SNP: Non Synonymous	Exon	Chr8	276669	G	A	G	A
*DTH8*	Os08g0174500	LOC_Os08g07740	-	-	-	-	-	-	-	-
*OsK4*	Os08g0484600	LOC_Os08g37800	-	-	-	-	-	-	-	-
*OsCO3*	Os09g0240200	LOC_Os09g06464	-	-	-	-	-	-	-	-
*Ehd2*	Os10g0419200	LOC_Os10g28330	-	-	-	-	-	-	-	-
*Ehd1*	Os10g0463400	LOC_Os10g32600	-	-	-	-	-	-	-	-
*OsMADS56*	Os10g0536100	LOC_Os10g39130	SNP: Non Synonymous	Exon	Chr10	20872414	T	G	T	G
			SNP: Non Synonymous	Exon	Chr10	20872469	G	C	G	C
			SNP: Non Synonymous	Exon	Chr10	20872638	A	G	A	G
*OsFKF1*	Os11g0547000	LOC_Os11g34460	-	-	-	-	-	-	-	-
*OsVIL2*	Os12g0533500	LOC_Os12g34850	-	-	-	-	-	-	-	-

### Development DNA markers for providing evidence of polymorphisms

**Fig 7:**
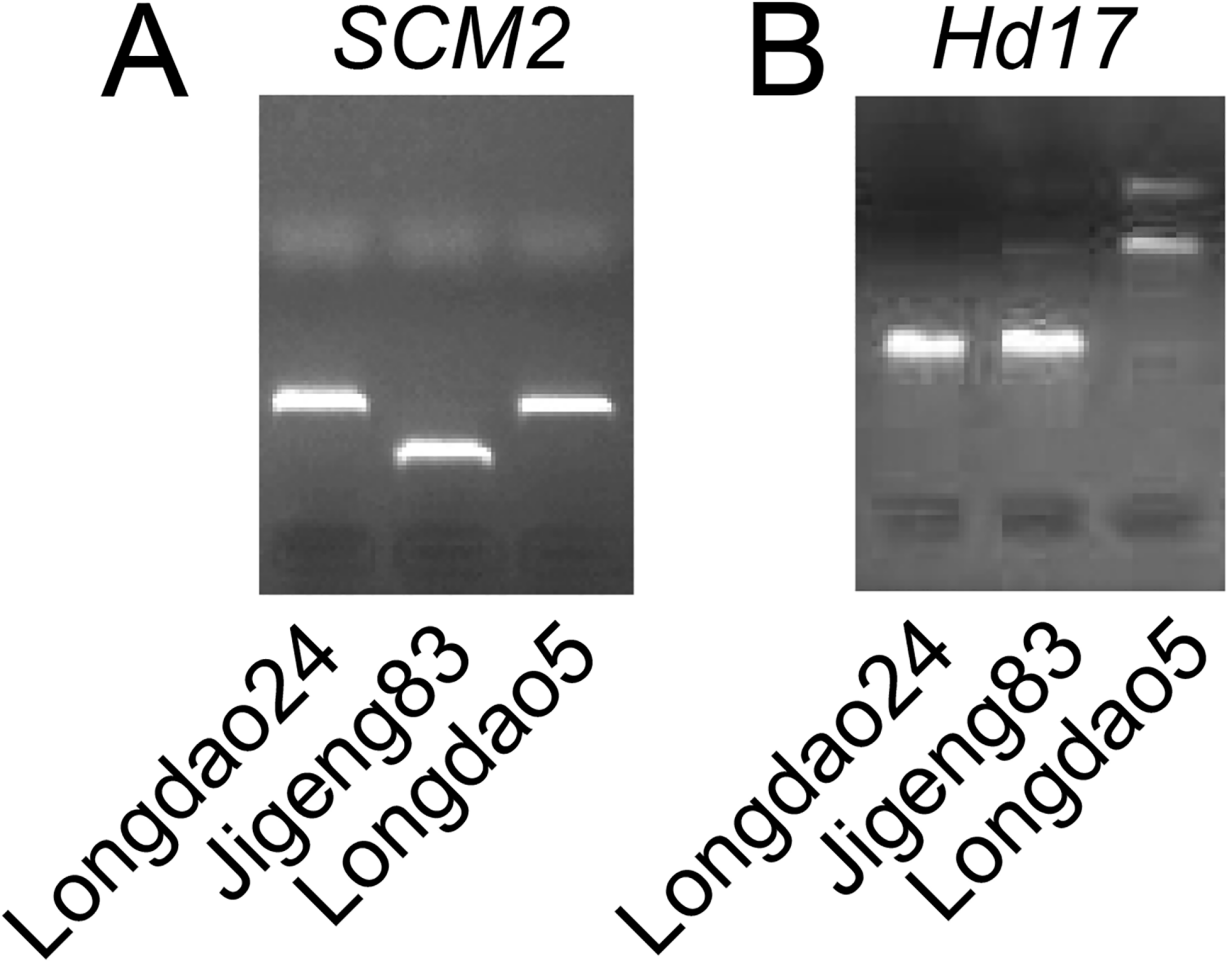
Development DNA markers for providing evidence of polymorphisms.

Although the accuracy of deep sequencing has now been greatly improved, it still needs to be confirmed by other methods for the variations of interest. Therefore, we selected one InDel and one SNP polymorphisms to developed DNA markers (Table 5). We designed the InDel marker according the 9 bp deletion in SCM2 locus. A complete matching genotypes was observed in Longdao24 and its parents. Longdao24 and Longdao5 was found having this 9-bp deletion compared to Jigeng83 (Fig 7A). We also developed a CAPS marker to provide the SNP (2235191) in Hd17 locus. A complete matching genotypes was also observed in Longdao24 and its parents (Fig 7B).

**Table 5:** The primers used for detection the polymorphisms in SCM2 and Hd17.

Gene	Marker type	Forward Prime	Reverse Primer	Enzyme
SCM2	InDel	CGACGGCGATGATGGAGG	CGGCCTGCTCGCGTTCCT	
Hd17	CAPS	CTACTCTTTGCCACCTGC	AAACAATATCGGATATTATAGC	Hpy166II

## Discussion

### Breeding value of the re-sequencing data of super high yield rice variety

Marker-assisted selection (MAS) is expected to provide higher efficiency, reduced cost and shorter duration of the breeding scheme, compared with conventional methods. But most important complex traits in rice, including yield, quality and stress tolerance, are controlled by quantitative trait loci (QTLs). Isolating and characterizing genes involved in QTLs has been the key step for MAS. Whole genome re-sequencing technology has provided the possibility of genome wide variation analysis in rice breeding varieties. Longdao24 had been used widely as breeding parent, because of its high yield, good quality, early heading date and strong stress tolerance. Genome sequencing of Longdao24 with 47 × coverage effective depth and 95.08% 10 × coverage ratio was achieved. A total of 420,475 SNPs, 95,624 InDels, and 14,112 SVs were yielded from the Longdao24 genome. We identified 361,117 SNPs and 81,488 InDels between Longdao24 genome and Longdao5 genome. We also detected 428,908 SNPs and 97,209 InDels between Longdao24 genome and Jigeng83 genome. These different variations will be used for developing new molecular markers for genetics research and MAS.

### The genetic basic of high yield in Longdao24

**Fig 8:**
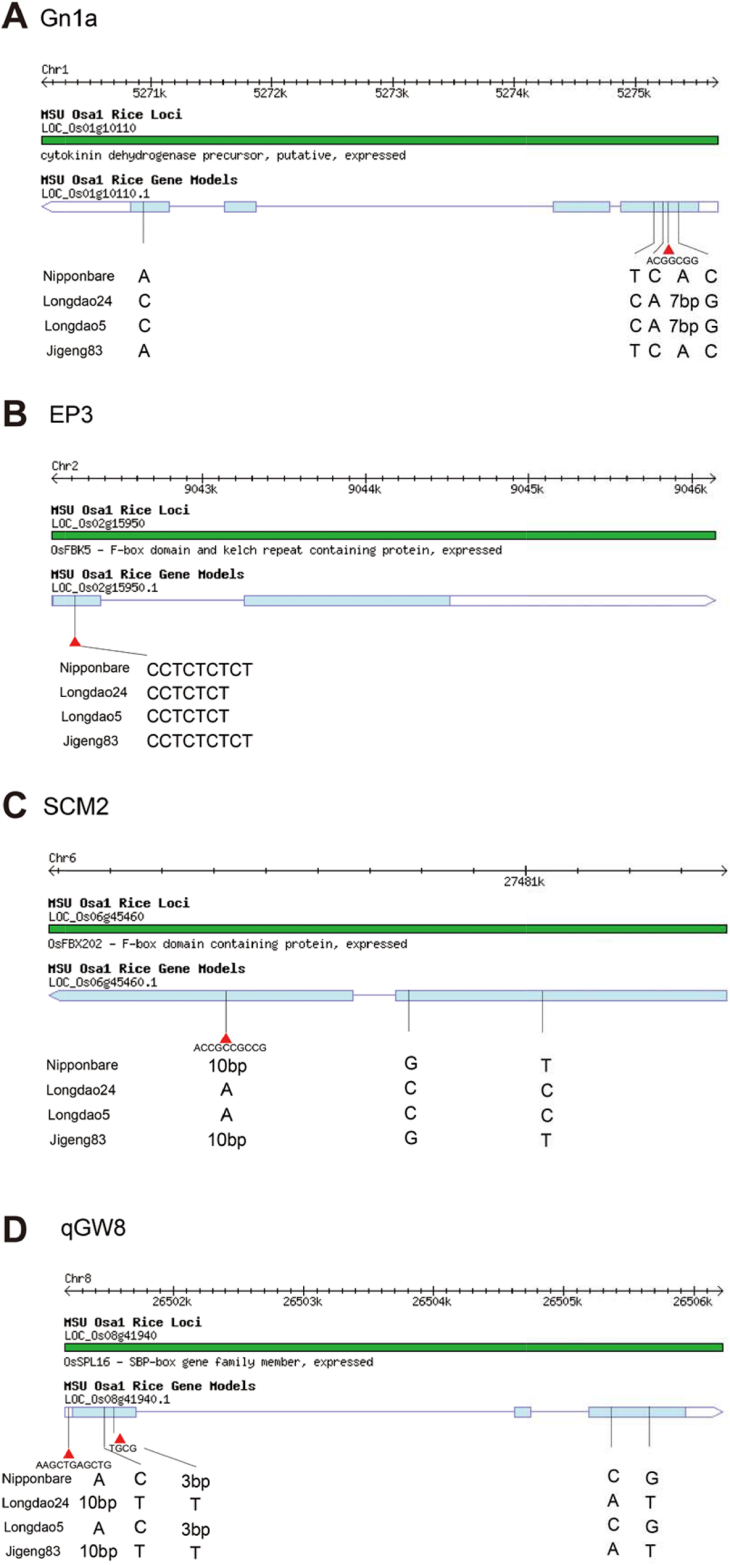
The yield related genes (*Gn1a*, *EP3*, *SCM2* and *qGW8*) having genetic variations in Longdao24.

Developments of whole genome re-sequencing technology have provided new tools for discovering and tagging novel useful alleles for improving target traits and for manipulating those genes in rice breeding program through MAS. We identified four yield related genes (*Gn1a*, *EP3*, *SCM2* and *qGW8*) having genetic variations in Longdao24. The first one is *Gn1a*, which controlled cytokinin accumulation in inflorescence meristems and increases the number of reproductive organs, resulting in enhanced grain yield [30]. Comparison of the DNA sequences between the cultivars revealed several nucleotide changes, including a 6-bp insertion in the first exon, and four nucleotide changes resulting in amino acid variation in the first and fourth exons of the Longdao24 and Longdao5 allele (Fig 8A). The *Gn1a* allele from Longdao24 and Longdao5 was similar to Habataki, which has been reported having the increasing *Gn1a* allele. The second one is *EP3*, which controlled panicle architecture and enhance the grain yield in rice [31]. Comparison of the DNA sequences between the cultivars revealed a 2-bp deletion in the first exon of the Longdao24 and Longdao5 allele (Fig 8B). The *EP3* allele from Longdao24 and Longdao5 was a novel allele. The third one is *SCM2*, which showed enhanced culm strength and increased spikelet number [32]. Comparison of the DNA sequences between the cultivars revealed several nucleotide changes, including a 9-bp deletion in the second exon, and two nucleotide changes resulting in amino acid variation in the first exons of the Longdao24 and Longdao5 allele (Fig 8C). The *SCM2* allele from Longdao24 and Longdao5 was same as Habataki, which has been reported having the increasing *SCM2* allele. The last one is *qGW8*, which showed enhanced culm strength and increased spikelet number [33]. Comparison of the DNA sequences between the cultivars revealed several nucleotide changes, including a 10-bp insertion in the first exon, a 3-bp deletion in the first exon, and three nucleotide changes resulting in amino acid variation in the first and third exons of the Longdao24 and Jigeng83 allele (Fig 8D). The *qGW8* allele from Longdao24 and Longdao5 was same as HJX74, which has been reported having the increasing yield haplotype. So, the super high yield formed by the large half erect panicle and lodging resistance of Longdao24 would be caused by these four increasing alleles of high yield genes including *Gn1a*, *EP3*, *SCM2* and *qGW8*.

### The genetic basic of early heading in Longdao24

**Fig 9:**
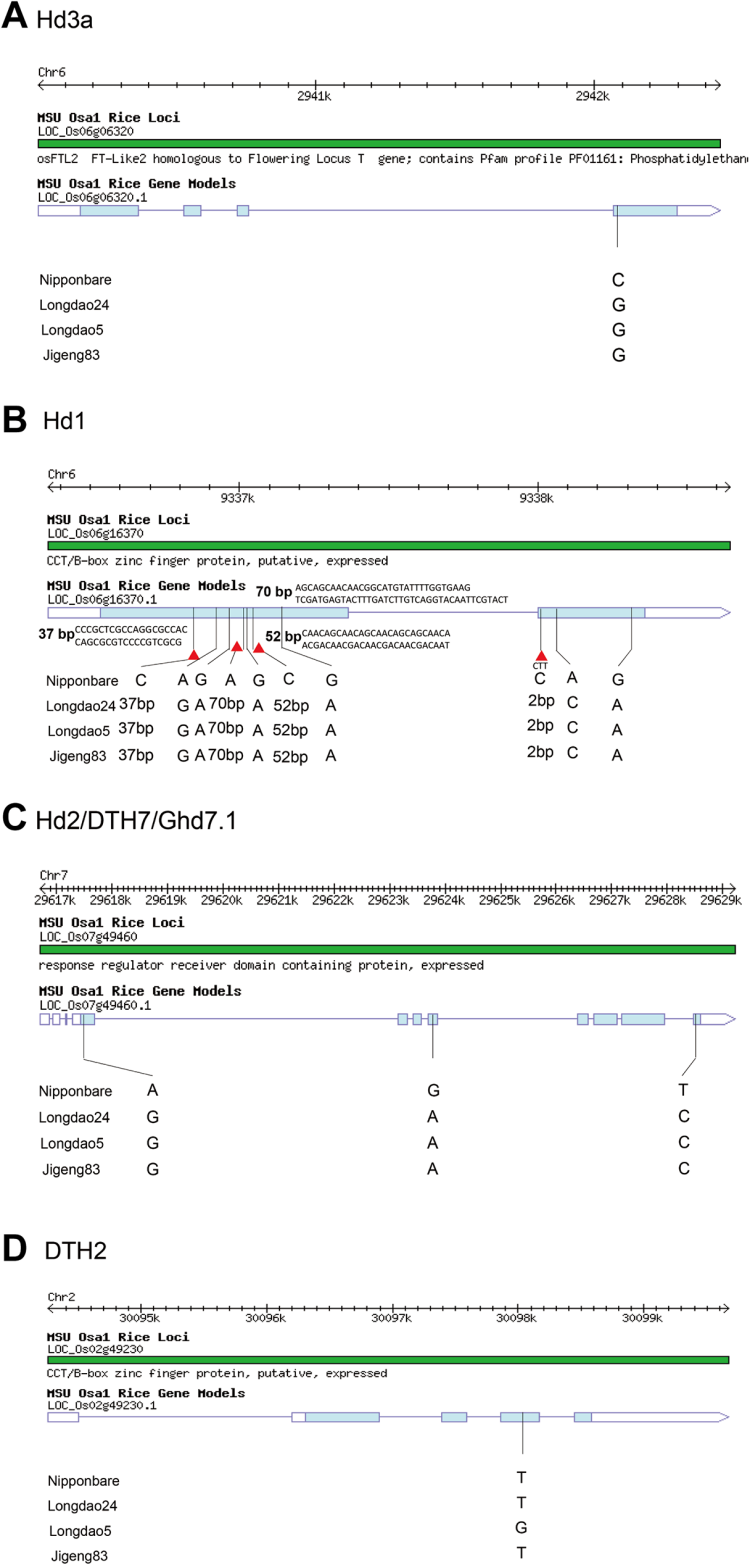
The heading related genes (*Hd3a*, *Hd1*, *Hd2* and *DTH2*) having genetic variations in Longdao24.

Heading date is a major determinant of rice adaptability to regional and environmental conditions. It has been a major selecting target in rice breeding programs. To elucidate the key heading date gene in Longdao24 for adaptation to the northern limit of rice cultivation Heilongjiang Province (43°26’-53°33’), we analyzed 39 cloned heading date related genes. *DTH2*, *OsLF*, *Hd17*, *Hd3a*, *Hd1*, *Hd2*, *Ehd3* and *OsMADS56* had variations among Nipponbare, Longdao24, Longdao5 and Jigeng83. The alleles of *Hd3a*, *Hd1* and *Hd2* in Longdao24, Longdao5 and Jigeng83 were different from that in Nipponbare. The might be the major reason for these three varieties adaptation to the northern limit of rice cultivation. *Hd1* and *Hd3a* are the two key genes controlled heading date in rice [34]. Comparison of the DNA sequences of *Hd3a* between the cultivars revealed only one nucleotide changes resulting in amino acid variation in the fourth exon of Longdao24, Longdao5 and Jigeng83 allele (Fig 9A). This *Hd3a* allele was a novel allele, which was not be reported before. The only one SNP caused an amino acid change at the carboxyl end of the predicted protein: the Asn (AAG) in Nipponbare was changed to Lys (AAC). The *Hd1* allele in Longdao24, Longdao5 and Jigeng83 was also a novel allele. Four InDels and six SNPs was identified by comparison of the DNA sequences of *Hd1* between the cultivars. Three long insertion and four nucleotide changes resulting in amino acid variation were identified in the first exon; and a 2-bp insertion and two nucleotide changes resulting in amino acid variation were identified in the second exon (Fig 9B). The *Hd2* allele in Longdao24, Longdao5 and Jigeng83 all had the three nucleotide changes resulting in amino acid variation in the first, fourth and eighth exons (Fig 9C). These variations were found in many European and Asian rice cultivars, which flower extremely early under natural long-day conditions [35]. The *DTH2* allele in Longdao24 was same as Jigeng83 (Fig 9D). This SNP in the third exon was reported as a key functional differences in the rice landraces from northern limit of rice cultivation in the world [36]. We also identified the allele of *Hd17*, *Ehd3* and *OsMADS56* in Longdao24 was came from Jigeng83 (Table 4). But only allele of *OsLF* was detected from Longdao5 (Table 4). All these information showed that the *Hd3a*, *Hd1* and *Hd2* might be the major reason for the varieties adaptation to the northern rice cultivation region. The *DTH2*, *OsLF*, *Hd17*, *Ehd3* and *OsMADS56* might formed different varieties adaptation to different region in northern rice cultivation location.

### The genetic basic of grain quality in Longdao24

Starch is the major component of rice grain, which is mainly composed of amylose and amylopectin. The starch synthesis pathway is an ideal system for examining the evolution of biochemical pathways. Over 20 genes involved in the rice starch synthesis pathway have been identified so far [37]. We investigated 22 cloned starch synthesis related genes in Longdao24 (Table 3). We only identified variations in *SSIVa*, *SBE3*, *SSIIIb* and *SSIIc* between the parents of Longdao24. All four allele of *SSIVa*, *SBE3*, *SSIIIb* and *SSIIc* were come from the male parent Jigeng83. These genes represented starch synthases (*SSIVa*, *SSIIIb* and *SSIIc*) and branching enzymes (*SBE3*). These enzymes are involved in amylopectin biosynthesis in rice endosperm. They had low to medium effects on variation in starch trait [38]. So we need do more works to get enough data, for example polymorphisms or expression levels of these genes, to explore the quality genetic basis of Longdao24.
